# Effect of maltodextrin on the oxidative stability of ultrasonically induced soybean oil bodies microcapsules

**DOI:** 10.3389/fnut.2022.1071462

**Published:** 2022-11-30

**Authors:** Zhimin Li, Bingyu Sun, Ying Zhu, Linlin Liu, Yuyang Huang, Mingshou Lu, Xiuqing Zhu, Yuan Gao

**Affiliations:** Key Laboratory of Grain Food and Comprehensive Processing of Grain Resource of Heilongjiang Province, College of Food Engineering, Harbin University of Commerce, Harbin, China

**Keywords:** soybean oil bodies, microencapsulation, maltodextrin, ultrasound, stability

## Abstract

**Introduction:**

Encapsulation of soybean oil bodies (OBs) using maltodextrin (MD) can improve their stability in different environmental stresses and enhance the transport and storage performance of OBs.

**Methods:**

In this study, the effects of different MD addition ratios [OBs: MD = 1:0, 1:0.5, 1:1, 1:1.5, and 1:2 (v/v)] on the physicochemical properties and oxidative stability of freeze-dried soybean OBs microcapsules were investigated. The effect of ultrasonic power (150–250 W) on the encapsulation effect and structural properties of oil body-maltodextrin (OB-MD) microcapsules were studied.

**Results:**

The addition of MD to OBs decreased the surface oil content and improved the encapsulation efficiency and oxidative stability of OBs. Scanning electron microscopy images revealed that the sonication promoted the adsorption of MD on the surface of OBs, forming a rugged spherical structure. The oil-body-maltodextrin (OB-MD) microcapsules showed a narrower particle size distribution and a lower-potential absolute value at an MD addition ratio of 1:1.5 and ultrasonic power of 250 W (32.1 mV). At this time, MD-encapsulated OBs particles had the highest encapsulation efficiency of 85.3%. Ultrasonic treatment improved encapsulation efficiency of OBs and increased wettability and emulsifying properties of MD. The encapsulation of OBs by MD was improved, and its oxidative stability was enhanced by ultrasound treatment, showing a lower hydrogen peroxide value (3.35 meq peroxide/kg) and thiobarbituric acid value (1.65 μmol/kg).

**Discussion:**

This study showed that the encapsulation of soybean OBs by MD improved the stability of OBs microcapsules and decreased the degree of lipid oxidation during storage. Ultrasonic pretreatment further improved the encapsulation efficiency of MD on soybean OBs, and significantly enhanced its physicochemical properties and oxidative stability.

## Introduction

Current research on soybean and its products mainly focus on the influence on the functional and structural characteristics of soybean protein while neglecting the critical component of the system-oil bodies (OBs). In soybean seeds, oil is mainly stored as triacylglycerols (TAG) in the subcellular organelles of the soybean oil bodies (1). Oil bodies (OBs) are spherical structures composed of TAG matrix, phospholipid monolayer and endogenous proteins (mainly oleosin) embedded in the phospholipid layer (2–4). The unique structure of OBs makes them physically and chemically stable against external environmental stresses such as heating, drying and freezing (5). In addition to TAG, OBs are also rich in polyunsaturated fat and has a small number of bioactive substances such as tocopherols, phytosterols, and soy isoflavones (6). Wang et al. (6) found that functional low-fat mayonnaise developed using soybean OBs as egg yolk substitutes not only had a lower cholesterol content but also released more antioxidant active ingredients during digestion. Soybean OBs are also used as natural carriers of polyphenols such as curcumin (7). Sun et al. (8) confirmed that embedding curcumin in OBs significantly improved curcumin’s solubility and chemical stability and enabled it to exhibit higher bioavailability. Therefore, OBs are widely used to develop functional foods and edible films.

Under certain conditions, especially near the oleosin isoelectric point, OBs exhibit significant aggregation and flocculation. At the same time, the presence of large amounts of polyunsaturated fatty acids in OBs makes them highly susceptible to lipid oxidation when exposed to light, oxygen, or moisture (9). To prevent this, OBs are frequently microencapsulated into the wall material to separate them from the outside environment and increase their stability (10). Maltodextrin (MD) is widely used in the microencapsulation of bioactive substances because of its high solubility, low viscosity at high solid concentration, low cost and neutral taste and color. In the packaging process, the wall material influences the physical and functional properties of microcapsules (11). MD can be used to protect core materials from external environmental damage and maximize the encapsulation of core materials (12). Fongin et al. (13) explained that adding MD during drying and storage can improve the microencapsulation efficiency and oxidation stability of the core material. Mohona and Pradyuman (14) revealed that MD was used effectively to prevent degradation and extend the bioactive substances’ shelf life in the encapsulation of fenugreek seed oil. Zhu et al. (15) found that the encapsulation efficiency of soybean OBs using maltodextrins with different degrees of hydrolysis reached up to 88.84 g/100 g, which significantly improved thermal and oxidative stability of OBs. Maurer et al. (16) found that the encapsulation efficiency and oxidative stability of the OBs were improved by adding maltodextrin. However, the emulsification performance of MD is poor, which seriously affects the physical properties of the cores (OBs).

Furthermore, other technologies, such as ultrasonic technology are needed to induce the microencapsulation of soybean OBs to obtain ideal microcapsule particles. As an emerging processing technology, ultrasound has received extensive attention in the food industry and has been successfully applied to preparing nanostructured carriers (17). In ultrasonic media, proteins are subjected to mechanical effects such as dynamic stirring, shear, turbulence, cavitation and thermal effects, resulting in changes in their physicochemical properties and protein structure. These altered properties facilitate the enhancement of protein-lipid and protein-polysaccharide interactions in OBs (18). Thus, ultrasound can be a potential processing technique for preparing biologically active OBs complexes. To the best of our knowledge, the effect of ultrasonic pretreatment on the physicochemical properties and oxidative stability of MD-encapsulated soybean OBs has not been reported.

Therefore, this study investigated and adopted a new method of preparing oil-body-maltodextrin (OB-MD) microcapsules by encapsulating soybean OBs in MD. The effects of the MD addition ratio on both encapsulation efficiency and oxidative stability of soybean oil bodies were studied. The structural characteristics of the microcapsules prepared using different ultrasonic powers were explored. The mechanism of encapsulation efficiency was elucidated and oxidative stability of OBs was analyzed by changes in structure.

## Materials and methods

### Experimental raw materials

Soybean was provided by Harbin Hagaoke Soybean Food Co., Ltd. Maltodextrin (DE = 19) was purchased from Henan Wanbang Industrial Co., Ltd. Sugar was purchased from Tianjin Tianli Chemical Reagent Co., Ltd. All other chemical reagents were of analytical grade.

### Experimental methods

#### Sample preparation

##### Extraction of oil bodies

The OBs were prepared using the method of Yeming and Tomotada (19) with slight modifications. The soybeans were soaked in deionised (DI) water (1:5, w/v) at 4°C for 18 h. The soaked soybeans were then ground in a tissue masher for 4 min, where the soybean to DI water bean to liquid ratio was 1:9 (w/v). After grinding, the insoluble material, such as soybean residue, was removed by filtration using triple skimmed gauze, and 20% (w/v) sucrose was added to the filtrate and stirred magnetically at 750 rpm for 30 min at 4°C. The pH of the solution was adjusted to 11.0 using 1 mol/L NaOH and centrifuged at 4°C and 8,000 × *g* for 30 min (Hunan Changsha Xiangyi Centrifuge Instrument Co., Ltd., Changsha, China). The upper layer of creamy OBs was collected and the excess water was blotted out with filter paper.

##### Preparation of oil-body-maltodextrin microcapsules

The preparation of OB-MD microcapsules was according to the method of Ding et al. (20) with slight modification. The OBs and MD solutions were prepared using DI water at a mass concentration of 20 mg/ml (w/v) and stirred magnetically at 750 rpm for 30 min at 4°C, respectively. The configured OBs were then mixed with the MD solution at a volume ratio (v/v) of 1:0, 1:0.5, 1:1, 1:1.5, and 1:2. All solutions were treated at an ultrasonic frequency of 40 Hz and power of 100, 175 and 250 W for 20 min (15D117, Ningbo Scientz Biotechnology Co., Ltd., Ningbo, China). The OB-MD without ultrasonic treatment was used as a blank control and the OB-MD powder was obtained after freeze-drying and fully ground. The different proportions of MD addition were named OBs, OB-MD 0.5, OB-MD 1, OB-MD 1.5, and OB-MD 2, respectively.

#### Determination of particle size and ζ-potential of oil-body-maltodextrin microcapsules

The particle size and ζ-potential of OB-MD were determined by dynamic light scattering using the method of Chunyan et al. (21), with slight modifications. OB-MD was dissolved in DI water to 1 mg/ml (w/v), and the particle size distribution was determined using a potential and particle size analyzer (Nano-ZS-90, Malvern Company, Malvern, UK). Temperature was set at 25°C, refractive indexs of the soybean oil and dispersant (water) were 1.47 and 1.33. The ζ-potential of the samples was determined by a Nano-ZS-90 Potentiometric Analyser.

#### Determination of the physicochemical property of oil-body-maltodextrin microcapsules

##### Calculation of encapsulation efficiency

The calculation of the OB-MD encapsulation efficiency was based on the method of Emad et al. (22), with minor modifications. The oil on the surface of OB-MD was removed using hexane. OB-MD was mixed with hexane at a ratio of 1:10 (w/v), shaken and mixed for 2 min at 25°C, then filtered, and the residue was collected and washed three times in duplicate. The filter residue was placed in a rotary evaporator at 60°C water bath to remove residual hexane, and the surface oil content of OB-MD was calculated according to the weight loss. The total fat content was calculated by Soxhlet extraction. The encapsulation efficiency of OB-MD was calculated as follows:


$$\mathrm{Encapsulation}\,\mathrm{efficiency}\,\left(g/100\,g\right)=\frac{\mathrm{Total}\,\mathrm{oil}-\mathrm{Surface}\,\mathrm{oil}}{\mathrm{Total}\,\mathrm{oil}}\times 100$$

##### Determination of moisture content

The moisture content of OB-MD was determined by the method of OB-MD Aliakbarian et al. (23), with slight modifications. Sample (1 g) was heated in a hot air oven (DHG-92003A; Shanghai Yiheng Technology Co. Ltd., Shanghai, China) at 105°C until constant weight was achieved. The moisture content of OB-MD was calculated as follows:


$$\mathrm{Moisture}\,\mathrm{content}\,\left(g/100\,g\right)=\frac{W_{1}-W_{2}}{W_{1}}\times 100$$

where w_1_ is total weight of sample and w_2_ is weight of dried sample.

##### Determination of wettability

The determination of the wettability of OB-MD is based on the method of Karrar et al. (24) with minor modifications. OB-MD microcapsules (0.5 g) were placed in 200 ml DI water at 25°C, and the time(s) required for the microcapsule powder to be thoroughly wetted was recorded.

##### Determination of hygroscopicity

The hygroscopicity of OB-MD was determined according to the method of Rafaella et al. (25). Microcapsule sample (1 g) was placed in a 25°C incubator (DHP-9162; Shanghai Yiheng Technology Co., Ltd., Shanghai, China), and the relative humidity inside the incubator was adjusted to 75.29% using saturated sodium chloride solution. Hygroscopicity of OB-MD was calculated as follows:


$$\mathrm{Hygroscopicity}\,\left(g/100\,g\right)=\frac{w_{2-}\,w_{1}}{w_{1}}\times 100$$

where w_1_ is the total weight of the sample; w_2_ is the sample weight after moisture absorption.

##### Determination of bulk density

The bulk density of OB-MD was determined based on the method of Zhu et al. (26), with slight modifications. OB-MD microcapsules (0.2 g) were placed into a 10 ml measuring cylinder and record the volume of the sample in the cylinder. Bulk density of the OB-MD microcapsules was calculated as follows:


$$\mathrm{Bulk}\,\mathrm{density}\,\left(g/c\,m^{3}\right)=\frac{S\,a\,m\,p\,l\,e\,w\,e\,i\,g\,h\,t}{S\,a\,m\,p\,l\,e\,v\,o\,l\,u\,m\,e}$$

#### Determination of emulsification of oil-body-maltodextrin

The emulsification activity (EAI) and emulsion stability (ESI) of OB-MD were determined by the method of Wenhui et al. (27) with some modifications. A solution of OB-MD at a mass concentration of 2 mg/ml (w/v) was prepared using DI water. The suspension was mixed with edible oil at a volume ratio of 3:1 (v/v) and homogenized for 2 min at 10,000 rpm using a high-speed disperser. From the dispersion, 50 μL was sampled at 5 mm from the bottom of the centrifuge tube at 0 and 30 min, respectively, and mixed with 5 ml of 1% (w/v) sodium dodecyl sulfate (SDS). The absorbance of the samples was measured at 500 nm using an A1pHa-1506 UV-VIS spectrophotometer (Shanghai Spectrum Instrument Co., Ltd., Shanghai, China) and recorded as A_0_ and A_30_, respectively. The EAI and ESI of the microcapsules were calculated as follows.


$$E\,A\,I\,\left(m^{2}/g\right)=\frac{2\times 2.303\times A_{0}\times D}{\rho \times \varphi \times 1000}$$

$$E\,S\,I\,\left(m\,i\,n\right)=\frac{A_{0\times \Delta \,t}}{A_{0}-A_{30}}$$

where D is dilution multiple, 250; ρ is sample concentration, g/ml and φ is the volume fraction of the oil phase, 0.25.

#### Determination of oil-body-maltodextrin protein components

The analysis of protein components in OB-MD was slightly modified based on the method of Li et al. (28). The protein fractions were separated by polyacrylamide gel electrophoresis (SDS-PAGE). The stacking gel and separating gel concentrations were 4 and 20%, respectively. OB-MD microcapsules prepared in section “Preparation of oil-body-maltodextrin microcapsules” were dissolved in 2% SDS solution to a final solubility of 3 mg/ml. The protein solution (80 μL) was mixed with 20 μL protein buffer solution (containing β-mercaptoethanol), heated in a boiling water bath for 5 min and centrifuged at 4,000 × *g* for 5 min. A pipette was used to inject 20 μL of treated sample into the injection port. The protein bands were separated at a constant voltage. The electrophoresis was run for 30 min at 80 V in the concentrated gel and about 70 min at 120 V in the separation gel. After electrophoresis, staining was performed using Coomassie brilliant blue R-250 equiped with a gel imaging system (EN-88; Seiko Epson Corp, Japan) which was used to observe and analyze the protein bands in OB-MD.

#### Determination of Fourier transform infrared spectroscopy

According to the method Zhang et al. (29), the chemical structure of OB-MD was separated by polyacrylamide gel electrophoresis (SDS-PAGE). Using an infrared spectrometer (Spectrum Two, PerkinElmer, Germany), the concentrated and separated gel concentrations were 4 and 20%, respectively. The temperature was set to 25°C, wavelength range was 400–4,000 cm^–1^, resolution was 4 cm^–1^, and the scan was 32 times. The secondary structure of the OBs-related proteins was analyzed by Peak fit Version 4.12 software. The percentages of α-helix, β-sheet, β-turn and random coil were calculated according to the Gaussian peak fitting algorithm.

#### Scanning electron microscopy analysis of oil-body-maltodextrin

The morphological and microstructural characteristics of soybean OB-MD were observed using scanning electron microscopy (SEM) (20). The microcapsule particles were attached to double-sided adhesive tape and a thin layer of gold was coated to the surface. The morphology of the microcapsules was observed under vacuum conditions at an accelerating voltage of 5 kV, and a magnification of 10 k was selected to capture digital images.

#### Determination of oil-body-maltodextrin oxidation stability

The oxidative stability of OB-MD was determined by accelerated oxidation experiments. The plates containing microcapsules were stored in an incubator at 60°C for 7 days. The primary and secondary oxidation products were analyzed daily to measure the degree of lipid oxidation.

##### Determination of hydroperoxide values

The primary oxidation product content was determined by measuring the peroxide value (PV), following the method of Yun and He (30) with minor modifications. Sample (0.5 g) was mixed with 15 ml of acetic acid-chloroform solution (1:3, v/v), shaken 10 times and centrifuged at 4,000 × *g* for 10 min. Supernatant (0.2 ml organic phase) was mixed with 2.8 ml of methanol-butanol solution (2:1, v/v), 15 μL of potassium thiocyanate solution (3.9 mol/L) and 15 μL of ferrous chloride solution (0.72 mol/L) for 20 min in the dark. The absorbance was measured at 510 nm using a methanol-butanol solution as a blank control, and the content of hydroperoxides was calculated from the Fe^3+^ standard curve.

##### Determination of thiobarbituric acid reactive substances

Malondialdehyde (MDA) in lipid peroxide degradation products can be condensed with thiobarbituric acid (TBA) to form red products with maximum absorption at 532 nm (31). Therefore, the oxidation degree of oil can be measured by measuring the TBA value of secondary oxidation products. TBA value of OB-MD was determined according to the method of Li et al. (32). Sample (0.5 g) was dispersed in 5 ml of DI water and mixed with 0.3 ml of the sample with 5 ml of trichloroacetic acid solution (100 g/L) and 2 ml of TBA (10 g/L) in a boiling water bath for 30 min. After cooling to room temperature, the supernatant was centrifuged at 6,000 × *g* for 15 min at 4°C. The absorbance at 532 nm was measured, and the thiobarbituric acid reactive substances (TBARS) content in soybean OBs was calculated according to the external standard curve of 1,1,3,3-tetra ethoxy propane.

#### Data processing

All results were measured in triplicate and expressed as mean ± standard deviation. The mean values were compared by S-N-K analysis (*P* < 0.05). Statistical package for social sciences (SPSS version 16.0) software was used for statistical analysis and data processing, and the images were processed using Origin 2018 software.

## Results and analysis

### Effect of ultrasound power on oil-body-maltodextrin particle size distribution

**FIGURE 1:**
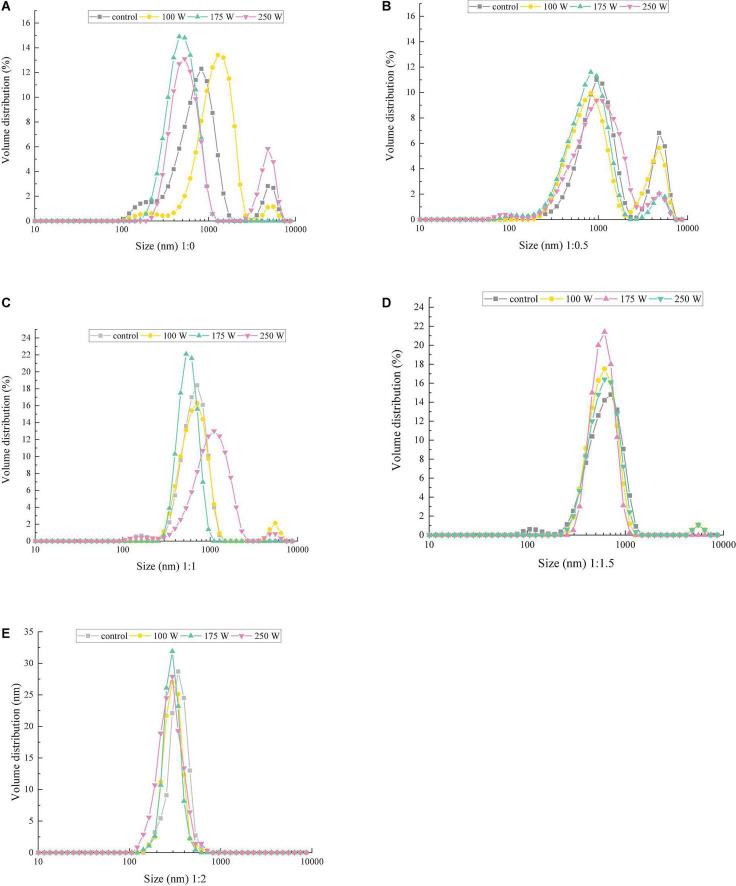
Effect of ultrasonic power on the particle size distribution of microcapsules with different ratios of OB–MD: **(A)** OBs, **(B)** OB-MD 0.5, **(C)** OB-MD 1, **(D)** OB-MD 1.5, and **(E)** OB-MD 2.

Particle size distribution significantly affects powder appearance, distributivity and fluidity, and food texture and sensory characteristics. Figures 1A–E shows the effect of MD addition and ultrasonic power on the particle size distribution of OB-MD. Addition of MD significantly affected the particle size of OB-MD. The OBs particles lacking wall material (MD) exhibited a precise three-peaked distribution, which indicated the presence of large aggregates in the pure OBs particles and can adversely affect the dispersion and stability of the emulsion. In contrast, MD significantly decreased the particle size of OBs, with the particle size distribution of OB-MD gradually shifting to a single-peaked distribution as the proportion of MD increased. When the volume ratio of OBs to MD was 1:2, the particles of OB-MD was the smallest, and the particle size distribution was the narrowest. This is because OBs undergo dehydration shrinkage during the drying process in the presence of MD, which decreases their particle size. Zhu et al. (15) reached similar conclusions: the smaller the OBs particles, the more homogeneous the particle size distribution of OB-MD. The above results show that when the volume fraction of the oil phase is too high, the excess oil cannot be well wrapped by the wall material. The excess fat forms larger oil droplets through the medium flow, resulting in larger particle sizes after freeze-drying.

In addition, it can be seen from Figure 1A that ultrasonic treatment significantly affects the particle size distribution of OBs. With increase in ultrasonic power, the particle size distribution of OBs near 150 and 800 nm gradually disappeared, while the particle size distribution near 500 nm gradually increased. When the ultrasonic power was 175 W, the particle size of OBs was the smallest, and the particle size distribution was the narrowest. Similar trends were also observed in microcapsules with different MD additions (Figures 1B–E). This may be due to the high-speed collision of OBs in the ultrasound medium during sonication and the increased fragmentation of droplets, which decreased particle size and increased dispersion of oil droplets in the system. Zhong and Xiong (33) found that cavitation and shear effects produced by ultrasonic treatment decreases the size of OBs droplets and increases the adsorbable area of the protein, thereby increasing the stability of the interfacial film around the oil droplets and the stability of the OBs emulsion.

### Effect of ultrasound power on ζ-potential of oil-body-maltodextrin microcapsules

**FIGURE 2:**
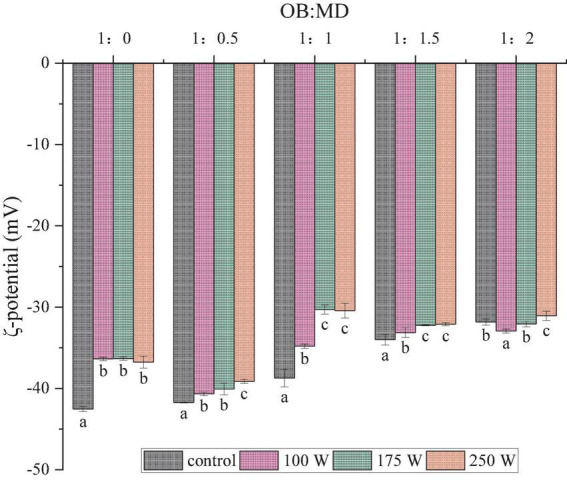
Effect of ultrasonic power on the ζ-potential of OB-MD microcapsules at different ratios. Different letters (a–c) in the figure indicate significant differences (*P* < 0.05).

The higher the absolute value of the ζ-potential, the greater the electrostatic repulsion between dispersed particles, and the less likely it is that collisions will result in aggregation (34). The effects of ultrasonic power and MD addition ratio on the microcapsules of OBs are shown in Figure 2. The maximum absolute value of ζ-potential of untreated OBs sample was 42.5 mV. The surface electrostatic charge of OBs decreased significantly after MD encapsulation, and the absolute value of ζ-potential decreased gradually as the proportion of MD increased (*P* < 0.05). This is similar to the findings of Ding et al. (20), where an increase in the absolute value of the ζ-potential was observed in the preparation of MD and chitosan-EGCG complexes encapsulating soybean oil bodies. MD is a neutral polysaccharide, whereas oleosin has a negatively charged surface, creating an interfacial film around the OBs according to electrostatic deposition theory (35). The decrease in the absolute value of ζ-potential after adding MD indicated that MD was successfully attracted to the surface of OBs by electrostatic deposition. In addition, it appeared that the absolute value of ζ-potential of OB-MD in solution decreased with the increase of ultrasonic power at the same MD addition ratio. Ultrasonic treatment amplified the electrostatic shielding effect of MD on OBs and decreased the net charge around the surface protein. The reduction in the net charge of OB-MD suggests that sonication promoted MD adsorption around oleosin and showed some frequency dependence. Ultrasonic treatment enhanced the encapsulation effect of MD on OBs. It promoted the shrinkage of soybean OBs in freeze-drying, which explains the reduction of OB-MD particle size by ultrasonication.

### Effect of ultrasonic power on physicochemical properties of oil-body-maltodextrin microcapsules

#### Effect of ultrasonic power on encapsulation efficiency of oil-body-maltodextrin microcapsules

**FIGURE 3:**
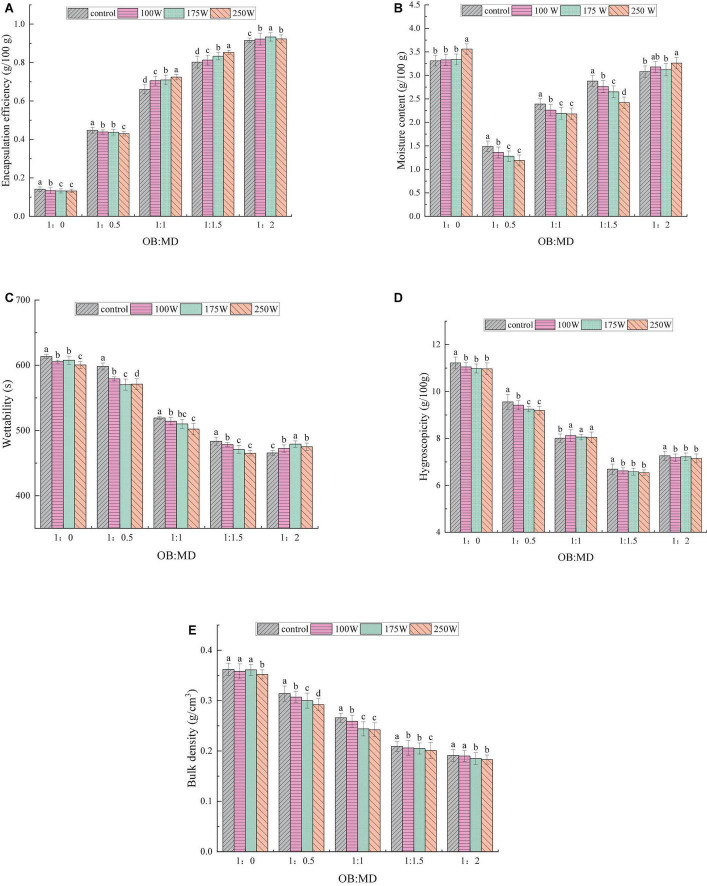
Effect of different dextrin additions and ultrasonic power on the physicochemical property of freeze-dried OB-MD microcapsules: **(A)** Encapsulation efficiency (%), **(B)** moisture content (g/100g), **(C)** wetting time (s), **(D)** hygroscopicity (g/100g), and **(E)** bulk density (g/cm^3^). Different letters (a–d) in the figure indicate significant differences (*P* < 0.05).

Encapsulation efficiency is an important indicator to evaluate the quality of microencapsulated products, and a higher encapsulation efficiency helps to avoid lipid oxidation in OBs. As shown in Figure 3A, the encapsulation efficiencies of OB-MD 0.5, OB-MD 1, OB-MD 1.5, and OB-MD 2 were 14.12, 44.77, 60.05, 80.21, and 91.55%, respectively. The encapsulation efficiency of OB-MD was significantly improved by adding MD. Karrar et al. (24) revealed that MD can effectively enhance the interfacial film on the surface of emulsified droplets, leading to a reduction in the oil content of the microcapsule surface after freeze-drying. During the preparation of OB-MD, ultrasonic treatment further improved the encapsulation efficiency of OBs, reaching a maximum of 93.33%. The particle size of the oil droplets decreased after ultrasonic treatment, and the contact surface area between the OBs emulsion droplets and MD increased. This improved the interaction between OBs and MD, and more MD were available to be wrapped on the surface of the oil droplets. The increase in encapsulation efficiency of OB-MD was also related to the increase in emulsifying activity of OBs. Wei and Gao (36) found that the microcapsules were easier to make by ultrasound treatment because the emulsifying activity of proteins improved, and the core and wall materials were better integrated into the solution.

Moreover, a certain thermal effect will be generated during the ultrasonic process, which promotes the glycosylation reaction of MD on the protein surface to a certain extent. The protein molecule’s covalent interaction between ε-amino (lysine) and the reducing carbonyl group at the end of the sugar molecule by Maillard reaction, might be a reason why the ultrasonication treatment improved the efficiency of MD encapsulation of OBs (37).

#### Effect of ultrasonic power on moisture content of oil-body-maltodextrin microcapsules

In terms of OB-MD, the moisture content is an essential indicator of its storage stability. The lower the moisture content of the microcapsules, the longer their storage time. The effects of MD addition ratio and ultrasonic power on the moisture content in OB-MD are shown in Figure 3B. Without ultrasonic treatment, the moisture content of OB-MD decreased from 3.22 to 1.49% (*P* < 0.05) with increase in MD addition ratio. When the ratio of OBs to MD was 1:1.5, the microcapsules had the lowest moisture content, indicating that MD successfully encapsulated the OBs and formed a dense protective layer around them, effectively preventing contact between oil and water in the air. This is consistent with the conclusion made by Ding et al. (20). In the drying process of microcapsules, the presence of wall materials can effectively promote the evaporation of water and decrease the water content of the powder after drying.

Interestingly, when the addition ratio of MD was 1:2, the moisture content of OB-MD increased significantly. The increase in the water content of OB-MD is related to the chemical structure of MD, which contains large number of hydrophilic groups side-linked and easily bound to water molecules in the air (38). Kang et al. (39) revealed that the wall material in microcapsules is mainly in the glassy state, which transforms into an amorphous rubbery state when the water content is too high, leading to the degradation and release of the core material. According to Emad et al. (22), when the moisture content of the microcapsules is less than 3 g/100 g, they have a remain stable over a long storage period. Therefore, the amount of MD added during the encapsulation of OBs should not be excessive. The ultrasonic power had a significant effect on the moisture content of the microcapsules. The moisture content of OB-MD decreased with increasing ultrasonic power (*P* < 0.05), with the lowest moisture content of the microcapsules at 250 W of ultrasound. The decrease in moisture content may be related to the increased encapsulation efficiency of MD on OBs by ultrasound treatment.

#### Effect of ultrasonic power on wettability of oil-body-maltodextrin microcapsules

Wettability and solubility reflect the reassembly properties of microcapsules. Wettability refers to the ability of the powder to absorb liquid under capillary action. Generally, powder with a short dissolution time in the water has good physical stability (40). The wetting time of OB-MD microcapsules is shown in Figure 3C. OBs had the longest wetting time of 613.37 s. The wetting time of microcapsules embedded with MD was shorter and faster than the control, and the wetting speed increased with increase in percentage of MD. The increase in wetting performance may be due to the lower oil content of the OB-MD surface and the smaller contact angle of the microcapsules with water. It was also found that sonication helped to increase the diffusion rate of OB-MD in water, where OB-MD 0.5, OB-MD 1, and OB-MD 1.5, and the diffusion time in water decreased with increasing sonication power. Badin et al. (41) reported that the reconstitution time of MD in the glassy state exhibited a strong frequency dependence. Sonication helped to prevent lump or precipitation of the powder and improved its wettability. The wettability of OB-MD 2 decreased with increase in ultrasonic power because the water content of microcapsules was high and it was easy to convert to a rubber state. Ultrasonic treatment accelerates the conversion rate of MD to a rubber state, which enabled MD form a dense layer on the surface of OBs and prevented the diffusion of OB-MD in water (42).

#### Effect of ultrasonic power on hygroscopicity of oil-body-maltodextrin microcapsules

Hygroscopicity is the ability of a solid powder to absorb moisture in the air, which can lead to the oxidation of lipids in the powder, which can affect its nutritional value and powder flow (26). To further evaluate the storage characteristics of OB-MD, its moisture absorption at a constant temperature and humidity environment was analyzed, and the results are shown in Figure 3D. The hygroscopicity of OB-MD was significantly decreased after MD encapsulation compared to OBs, with OB-MD 1.5 (OB: MD = 1: 1.5) exhibiting the lowest hygroscopicity of 6.69 g/100 g (*P* < 0.05). The ultrasonic treatment decreased the hygroscopicity of OB-MD. The change in hygroscopicity is related to the reduction of the moisture content of the powder. During the drying process, MD absorbed most of the water in OBs and formed a dense protective shell on its outside layer, effectively reducing the entry of external moisture (43).

#### Effect of ultrasonic power on bulk density of oil-body-maltodextrin microcapsules

Bulk density is an essential factor that affects packaging, transport and storage of powders. The bulk density of soybean OBs microcapsules are shown in Figure 3E. Bulk density of OB-MD decreased from 0.362 to 0.191 g/cm^3^ as the percentage of MD addition increased, with OB-MD 2 (OB: MD = 1: 2) exhibiting the lowest bulk density (*P* < 0.05). The ultrasonic treatment further decreased the bulk density of the microcapsules, with a minimum of 0.183 g/cm^3^. The above results indicate that the wall material ratio and ultrasonic treatment greatly influenced the bulk density of OB-MD. The lowest bulk density of OB-MD 2 may be due to the nature of the microcapsule wall material formed by MD. The high proportion of MD in the particles significantly affected their bulk density. Particle size of microcapsules is another major factor affecting bulk density. The larger the particle size, the more inconsistent the shape, the wider the gap between the particles and the lighter the mass per unit volume (44). As a result, when the size of the microencapsulated particles increased, their stacking density also decreased. Chew et al. (40) found that the stacking density of microcapsules decreased with increasing particle size, which is consistent with the findings of this study.

### Effect of ultrasonic power on emulsification characteristics of oil-body-maltodextrin microcapsules

**FIGURE 4:**
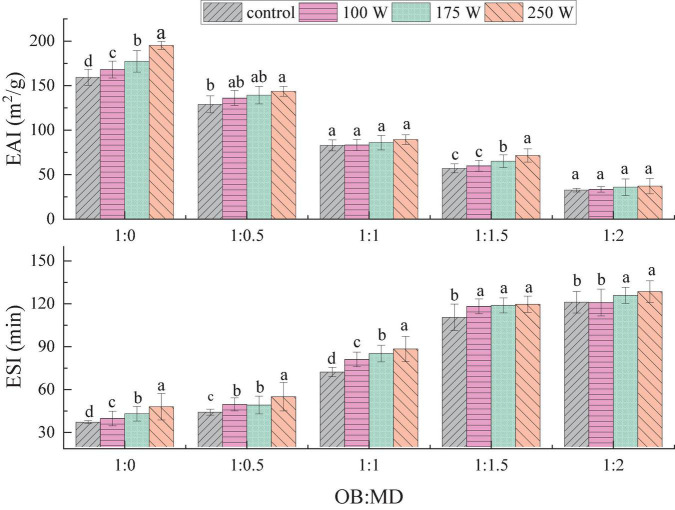
Effect of dextrin addition and ultrasonic power on the emulsification activity (EAI) and emulsion stability (ESI) of OB-MD. Different letters (a–d) in the figure indicate significant differences (*P* < 0.05).

Oil bodies can form natural O/W emulsions in solution, and the emulsification characteristics of OBs are essential indicators for evaluating their storage stability. The effects of ultrasonic power and MD addition ratio on the EAI and ESI of OBs and OB-MD microcapsules are shown in Figure 4. As MD embedding ratio increased, EAI of OB-MD gradually decreased, and the ESI gradually increased, which was related to the lower EAI of MD. Ultrasonic treatment had a positive effect on the EAI and ESI of OBs. Compared with the OBs emulsion without ultrasonic treatment, EAI and ESI of OBs increased significantly with ultrasonic power (*P* < 0.05). When the ultrasonic power was 250 W, the emulsifying activity of OBs reached the maximum, EAI was 195.29 m^2^/g and ESI was 43.10 min. The enhanced emulsifying activity of OBs is related to their surface activity and adsorption at the oil-water interface. The surface-adsorbed proteins in OBs de-folded after sonication, leading to the exposure of the hydrophobic amino acids and an increase in molecular flexibility (18). The interaction between protein and phospholipid formed a stable emulsion membrane, which was uniformly wrapped around the oil droplets, thereby preventing flocculation and deposition of oil droplets. Thus, EAI and ESI of OB-MD microcapsules were significantly improved which is in agreement with the finds of O’Sullivan et al. (45).

### Effect of ultrasonic power on protein components in oil-body-maltodextrin microcapsules

The surface proteins of OBs play an essential role in maintaining the stability of OBs and regulating the size of oil droplets (46). There are two types of proteins in OBs endogenous proteins, and OBs adsorption proteins (9). The endogenous proteins intrinsic to OBs include oleosin, OBs calmodulin, and OBs sterolins. While grinding soybean seeds, proteins stored in plant protein storage vacuoles are released and then adsorbed onto the surface of OBs as adsorption proteins (47). Among them, oleosin is the main protein in OBs, which mainly contains 24, 18, and 16 kDa components, and the component of 16 kDa is the least (5). OBs adsorption proteins are proteins adsorbed from the environment during the preparation of OBs, including soybean globulin (11S, including an acidic subunit A and an alkaline subunit), β-conglycinin (7S, including α, α′, and β subunits) and some enzymes (lipoxygenase, phospholipase, and protease) (48).

**FIGURE 5:**
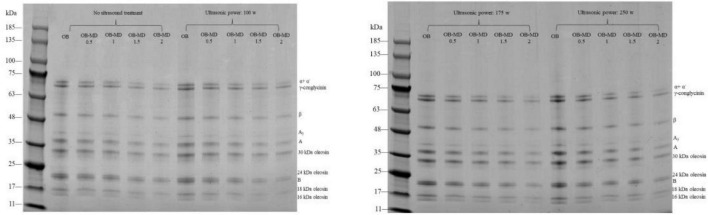
SDS-PAGE pattern of surface proteins in microencapsulated soybean OBs.

A total of 10 protein bands were found as shown in the SDS-PAGE results of proteins in OB-MD microcapsules shown in Figure 5. The same protein bands were obtained in OBs extracted by Qiying and Yufei (49), indicating that there were not only adsorbed proteins in OBs but also endogenous proteins. It can be seen from Figure 5 that ultrasonic treatment did not destroy the subunit structure of oleosin and OBs adsorbed protein in OBs. Ultrasonic treatment at a certain power did not change the primary structure of the OBs protein. The experimental results of ultrasonic treatment on the change of oleosin component are consistent with Sha and Xiong (50) and Zhang et al. (51). However, the exogenous protein content in OBs was significantly higher, and the intensity of the 7S and 11S protein bands increased after the sonication treatment. This is because the rearrangement of the molecular structure of soy protein and the content of exogenous proteins adsorbed around OBs can be increased by sonication (52). Sun et al. (8) found that during ultrasonic treatment, cavitation and turbulence effects changed the charge distribution of proteins on the surface of OBs, promoted the adsorption of exogenous proteins on the surface of OBs and led to an increase in the concentration of interfacial proteins. The increase in exogenous protein concentration promoted interaction between MD and OBs. MD was more easily adsorbed on the surface of OBs. According to the particle size and potential change data, ultrasound promoted the dissociation and molecular reconstruction of protein in OBs, which was beneficial to the adsorption of MD on the surface of OBs, thus improving the encapsulation efficiency of OBs.

### Effect of ultrasonic power on the secondary structure of oil-body-maltodextrin microcapsules

**FIGURE 6:**
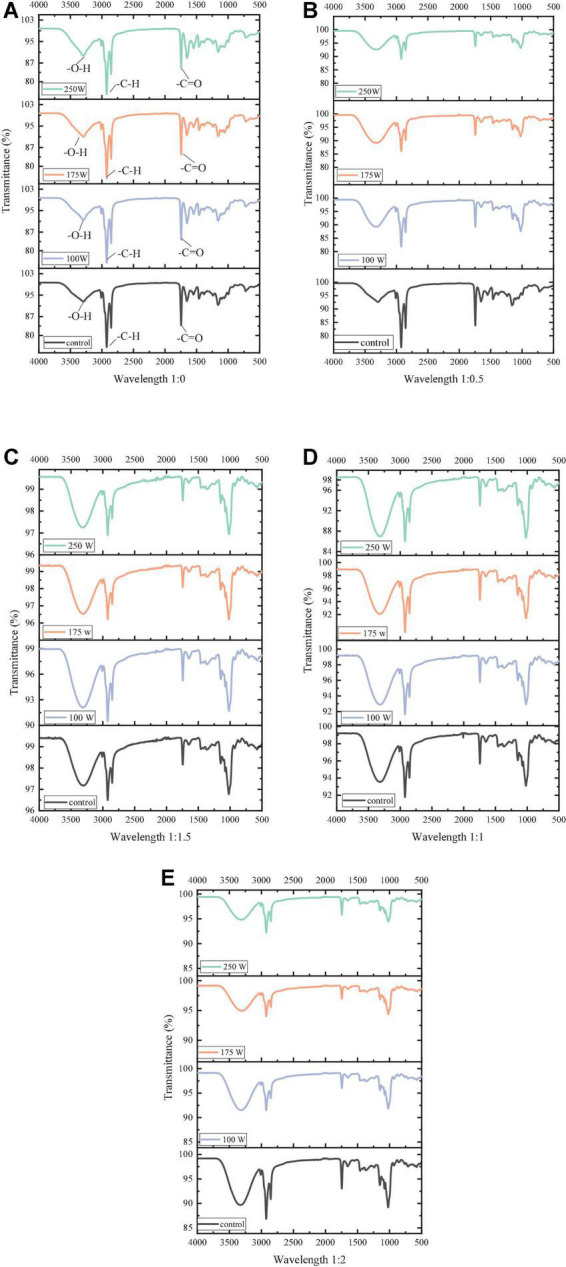
FTIR spectra of OB-MD at different MD addition ratios and ultrasonic power. **(A–E)** Functional groups in a wavelength range.

The interaction between maltodextrins and OBs and changes in the secondary structure of OBs-associated protein were further analyzed by measuring the Fourier transform infrared spectroscopy (FTIR) of OB-MD. From Figures 6A–E, it is observed that OBs and OB-MD had similar characteristic absorption peaks. Among them, the broad absorption band near the wave number of 3,300 cm^–1^, which is the characteristic absorption peak of -OH, the characteristic absorption peak of -CH at 2,940 cm^–1^, and the stretching vibration of -C=O double bond in triglyceride near 1,740 cm^–1^ (53). The above results show intramolecular solid and intermolecular hydrogen bonds (-OH stretching vibration) and hydrophobic bonds (-CH stretching vibration) in OBs and OB-MD. These results are consistent with the findings of Chew et al. (40). It indicated that oleosin was successfully adsorbed on the TAG matrix and bound to the triglyceride surface through hydrophobic interaction (54). As the proportion of maltodextrin increased, the peak area and intensity of the -OH absorption peak gradually increased. This is because MD is a macromolecular polymer linked by α-1,4 glycosidic bonds, and there is a large amount of -OH in its molecule (55). In addition, the intensity of the absorption peak at 2,940 cm^–1^ in the FTIR pattern of OB-MD gradually decreased with increasing percentage of MD, indicating that OBs were encapsulated by MD and bound to the surface of OBs through hydrophobic interaction (56). At the same MD addition ratio, the absorption intensity of the -CH absorption peak decreased with increasing ultrasound power, indicating that ultrasound treatment could promote the encapsulation of OBs by MD, which is consistent with the results of the encapsulation efficiency of OBs.

The absorption peak at 1,700–1,600 cm^–1^ in the FTIR spectrum represents the amide I band, indicating the secondary structural composition of the protein. The changes in the secondary structure of the adsorbed proteins in the OBs were further analyzed using a Gaussian fit, and the results are shown in Table 1. The α-helix of proteins in OBs was significantly decreased, while β-folding and β-turning angles were significantly increased after ultrasound treatment (*P* < 0.05). The ultrasound treatment promoted the unfolding of polypeptide chains in the protein structure, exposing the hydrophobic subunits inside. The α-helix content in the protein structure is negatively correlated with surface hydrophobicity. As the amount of α-helix decreased, the surface hydrophobicity of OBs surface proteins increased, which makes it easier for proteins to bind hydrophobically with MD *via* hydrogen bonding (53). At the same time, the increase in surface hydrophobicity also facilitates the adsorption of oil-body proteins at the O/W interface and enhances their emulsification capacity (57).

**TABLE 1:** Changes in the relative content of the secondary structure of the adsorbed protein in OBs under different ultrasonic power treatments.

	α -helix (%)	β -sheet (%)	β -turn (%)	Random curl (%)
Control	22.24 ± 1.12^a^	30.44 ± 0.15^c^	27.49 ± 1.21^b^	19.82 ± 0.74^a^
100 W	21.79 ± 0.98^b^	31.26 ± 0.11^b^	27.27 ± 1.05^c^	19.66 ± 0.67^b^
175 W	21.38 ± 0.11^c^	31.21 ± 0.75^b^	27.46 ± 1.15^b^	19.42 ± 0.12^c^
250 W	20.87 ± 0.79^d^	31.61 ± 0.12^a^	28.48 ± 0.96^a^	19.04 ± 0.78^d^

Mean ± standard deviation (*n* = 3); different letters (a–d) in each column indicate significant differences (*p* < 0.05).

### Effect of ultrasonic power on microstructure of oil-body-maltodextrin microcapsules

**FIGURE 7:**
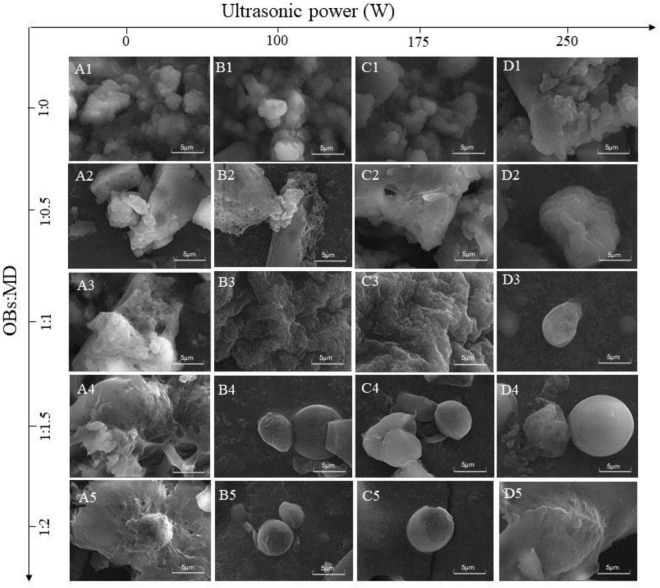
SEM images of OBs, OB-MD 0.5, OB-MD 1, OB-MD 1.5, and OB-MD 2 after different ultrasound power treatments. **(A1–A5)** No ultrasound, **(B1–B5)** 100 W ultrasound, **(C1–C5)** 175 W ultrasound, and **(D1–D5)** 250 W ultrasound.

The morphology of soybean OBs microcapsules was observed by SEM, and the results are shown in Figure 7. Among them, Figures 7A1–5 shows the morphological characteristics of the microcapsules formed by MD and OBs in a natural binding state without ultrasonic treatment. The change in the MD addition ratio resulted in different morphological characteristics of the freeze-dried microcapsules. OBs without MD encapsulation revealed depressions and cracks on the surface and irregular agglomeration, which may be related to the presence of large amounts of water on the surface of OBs and high levels of surface oil (58). After adding the wall material, the agglomeration of the oil droplets was decreased, and the surface became smooth and flat. In addition, larger lamellar structures were observed around the oil droplets, which were broken microcapsule walls. This indicates that OBs are not well encapsulated by MD when the loading of oil droplets in the system is too high. As the MD addition ratio increased, the agglomeration of OBs gradually disappeared. When MD addition ratio was 1:1.5 and 1:2, MD gradually adhered to the surface of OBs and formed an interfacial layer. These images suggest that MD formed a loose protective barrier around the soybean OBs, effectively preventing aggregation between oil droplets and resulting in a more desirable particle morphology (43). Compared with uncoated OBs, MD significantly improved the encapsulation efficiency of OBs, producing a more uniform surface coating and smaller particle size. Naqiyyah et al. (59) found that microencapsulated avocado seed oil had a smoother surface and smaller particle size.

Ultrasonication had a significant effect on the microscopic morphology of OB-MD. Ultrasonic treatment promoted the encapsulation of OBs by MD, resulting in a higher integrity microcapsule wall and a more homogeneous and stable morphology of the MD-encapsulated OBs after freeze-drying. Compared with the control, ultrasonic treatment further improved the encapsulation of OBs by MD, resulting in a complete microcapsule wall. When the MD addition ratio was 1:1.5, and the ultrasonic power was 250 W, the OB-MD microcapsules had the smoothest surface and the complete spherical structure. This has implications for producing homogeneous and stable microcapsules to avoid deterioration of soybean OBs caused by oil oxidation and uncontrolled oil spillage.

### Effect of ultrasonic power on oxidation stability of oil-body-maltodextrin microcapsules

**FIGURE 8:**
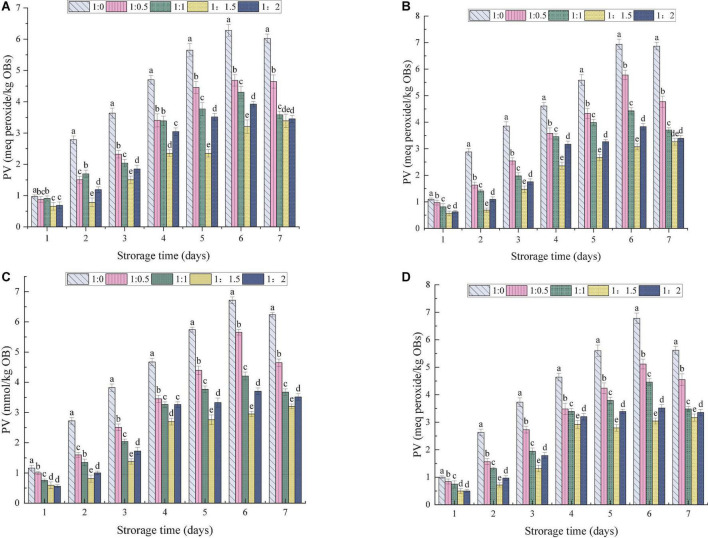
Effect of different dextrin addition and ultrasonic power on PV value of OB-MD: **(A)** no ultrasound, **(B)** ultrasonic 100 W, **(C)** ultrasonic 175 W, and **(D)** ultrasound 250 W. Different letters (a–e) in the figure indicate significant differences (*P* < 0.05).

**FIGURE 9:**
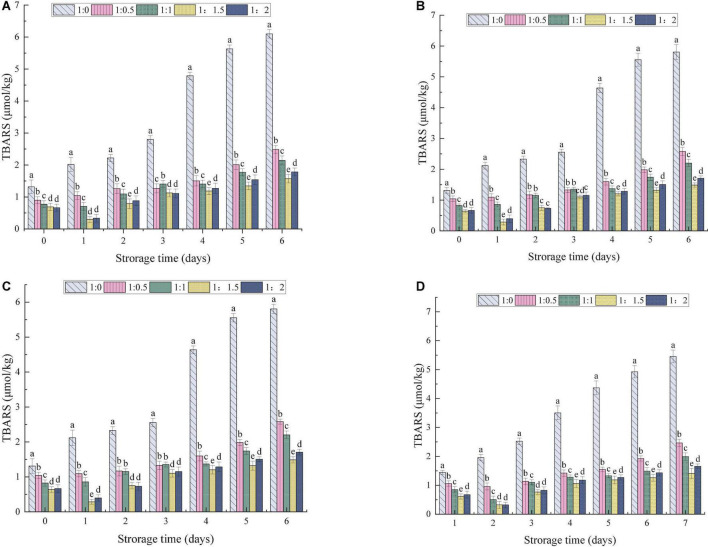
Effect of different dextrin addition and ultrasonic power on TBARS value of OB-MD: **(A)** no ultrasound, **(B)** ultrasonic 100 W, **(C)** ultrasonic 175 W, and **(D)** ultrasound 250 W. Different letters (a–e) in the figure indicate significant differences (*P* < 0.05).

The process of lipid oxidation usually produces aldehydes, ketones and other substances, leading to reduction in the quality of OBs and the production of offensive substances. At the same time, lipid oxidation also poses health risks (57). The effects of the MD addition ratio and ultrasonic power on the oxidative stability of OBs were evaluated. The amount of primary and secondary oxidation products produced by OB-MD during storage was reflected by PV and TBARS values, respectively. As shown in Figures 8, 9, the PV and TBARS values of the OBs without coating protection increased significantly throughout the storage period, with hydroperoxide values increasing from 0.96 to 6.28 meq peroxide/kg OBs and TBARS values increasing from 1.32 to 6.02 μmol/kg. The above results suggest that OBs were highly oxidized during storage, which is related to the high surface oil content of OBs and the lack of protective coating, thus predisposing them to induce free radical (hydroperoxide) formation during processing and storage (60). MD significantly protected OBs and the microencapsulated OB-MD had better oxidative stability than OBs. The degree of oxidation of oils and fats in OBs encapsulated by MD was significantly decreased, with significant differences in hydroperoxide and TBARS values during storage for OB-MD with different MD addition ratios (*P* < 0.05). This is related to the good antioxidant properties of MD, which adheres to the surface of OBs by microencapsulation technology and forms a dense protective barrier, effectively insulating them from oxygen and moisture in the air. This is consistent with the findings of Ling et al. (61) that the use of maltodextrin to encapsulate highly bioactive astragalus-like substances produced a more stable nanocomposite structure that could effectively improve its thermal, light, and storage stability. The PV of OB-MD 1.5 prepared by ultrasonication at 0, 100, 175, and 250 W were 3.38, 3.26, 3.20, and 3.16 meq peroxide/kg OBs and TBARS values were 1.57, 1.48, 1.48, and 1.40 μmol/kg, respectively. Compared to the non-sonicated microcapsules, OB-MD 1.5 had a better oxidative stability. The oxidative stability of the OB-MD prepared by 250 W sonication was better than that of the non-sonicated microcapsules. Thus, when ultrasonication was applied to microencapsulated soybean OBs, it effectively decreased lipid oxidation and extended the storage time of OBs.

### Formation mechanism of oil-body-maltodextrin microcapsules induced by ultrasound

**FIGURE 10:**
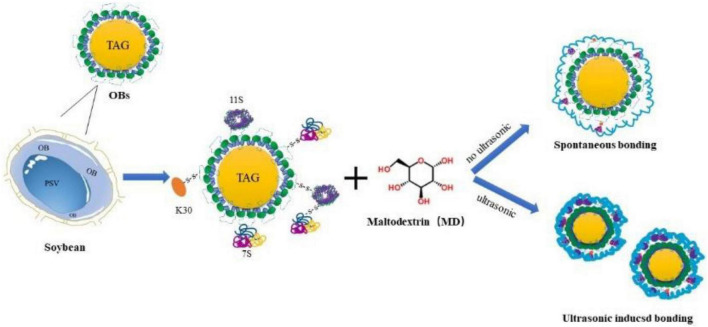
Formation mechanism of MD-embedded soybean OBs induced by ultrasound.

The formation mechanism of OB-MD induced by ultrasound is shown in Figure 10. The OB-MD non-covalent complexes can be produced by mixing MD solution and OBs solution with different concentrations. The complexation between MD and OBs occurs through hydrogen bonding, electrostatic interaction, and hydrophobic interaction, in which hydrophobic interaction is the main driving force (62). In the natural state, MD was adsorbed to the surface of OBs through non-covalent interactions and forms a hard “sugar shell” during drying. It forms a protective barrier against oxygen and moisture from the external environment, preventing oxidation and deterioration of the fat, thus maintaining the stability of the OBs. Ultrasonic treatment significantly improved the encapsulation efficiency of OBs. The higher the ultrasonic power, the smaller the particle size of the OBs produced, which increased the contact surface area between OBs and MDs and facilitated the adsorption of MDs on their surfaces. The interaction with OBs is actually the interaction with proteins on the surface of OBs. In the ultrasonic treatment process, oleosin was subjected to cavitation and turbulence effects, producing a strong force and leading to protein conformational changes. With increase in ultrasonic power, the structure of oleosin gradually unfolded and exposed a large number of hydrophobic amino acids, which promoted the interaction between MD and OBs. More MD were bound to the surface of OBs through hydrophobic interaction. At the same time, the ultrasonic treatment also promoted the dissociation and molecular reconstruction of exogenous proteins, increasing the content of adsorbed proteins on the surface of OBs, thus providing more active sites for MD adsorption. In summary, ultrasound treatment promoted the microencapsulation process of MD, enhanced the encapsulation efficiency of MD on OBs, and MD was tightly bound to OBs and formed a dense protective barrier, thus enhancing the protection of soybean OBs.

## Conclusion

As an ideal microcapsule wall material, MD can effectively protect soybean OBs from the pressure from external environment and has a significant impact on its physicochemical properties and oxidative stability. The proportion of wall material significantly affects the encapsulation efficiency of OBs, and it has been found that MD-coated OBs were more stable than free OBs. Ultrasonication significantly improved the encapsulation effect and emulsification properties of OBs. Ultrasonication promoted the adsorption of exogenous proteins on the surface of OBs. The mechanical effect changed the secondary structure of the proteins on the protein-phospholipid membrane surrounding OBs. Treatment of OBs and MDs pre-mixed solutions (OB: MD = 1: 1.5) by 250 W ultrasonic power resulted in OB-MD microcapsules with the best encapsulation efficiency and physicochemical properties and the highest oxidative stability. Ultrasonic treatment promoted the unfolding of oleosin structure and the change of secondary structure. The of α-helix content decreased and β-sheet and β-turn contents increased, which promoted inter and intramolecular hydrogen bonding reconstruction of proteins and the enhancement of hydrophobic interactions between MD and OBs, thus improving the encapsulation efficiency of MD on OBs. In general, ultrasonic treatment can be used as a promising technology to promote the microencapsulation of OBs, producing a new OBs product with good stability and emulsifying properties. This study provides a theoretical basis for applying ultrasonic technology in the delivery system of microencapsulated functional food and drug components.

## Data availability statement

The original contributions presented in this study are included in the article/Supplementary material, further inquiries can be directed to the corresponding authors.

## Author contributions

ZL: data curation, writing—original draft preparation, and review and editing. BS: methodology. YZ: resources. LL: formal analysis. YH: validation and visualization. ML: software and supervision. XZ: project administration and funding acquisition. All authors have read and agreed to the published version of the manuscript.
