# Supplementary figures and images for: Effect of maltodextrin on the oxidative stability of ultrasonically induced soybean oil bodies microcapsules
Source: Front Nutr. 2022 Nov 30;9:1071462. doi: 10.3389/fnut.2022.1071462 (PMC9748341; doi:10.3389/fnut.2022.1071462)

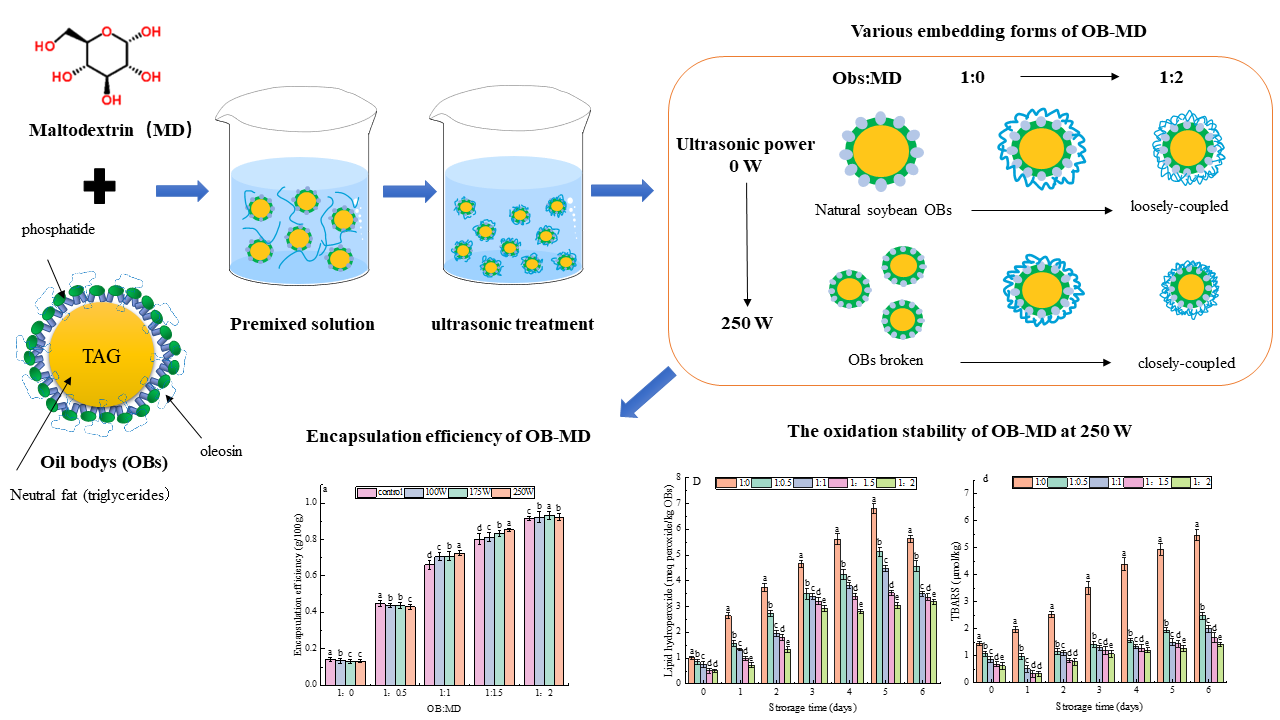

Supplement: Supplementary file 1 [file Image_1.PNG]
